# Does tobacco expenditure influence household spending patterns in Ghana?: Evidence from the Ghana 2012/2013 Living Standards Survey

**DOI:** 10.18332/tid/120936

**Published:** 2020-06-01

**Authors:** Abdul Gafar A. Masa-ud, Grieve Chelwa, Corné van Walbeek

**Affiliations:** 1 School of Economics, University of Cape Town, Cape Town, South Africa; 2 Graduate School of Business, University of Cape Town, Cape Town, South Africa

**Keywords:** tobacco, crowding-out, household expenditure

## Abstract

**INTRODUCTION:**

There is a growing literature on the ‘crowding-out’ effects of tobacco expenditure, particularly in Low-to-Middle Income Countries (LMICs). However, there is no published study investigating these effects in the context of Ghana, a country where tobacco consumption is expected to increase in the future. This study aims to investigate whether tobacco influences expenditure patterns within Ghanaian households.

**METHODS:**

We estimate a demand system of quadratic conditional Engel curves for a set of twelve groups of commodities using the 2012/2013 Ghana Living Standards Survey. Unlike previous studies we use the GMM 3SLS estimator, which provides more efficient parameter estimates due to heteroskedastic errors inherent in cross-sectional datasets of this nature.

**RESULTS:**

The results show that Ghanaian households that spend on tobacco are more likely to spend also on alcohol, recreation, transport and communications, but less on food, housing, and health needs.

**CONCLUSIONS:**

Tobacco expenditure, through its ‘crowding-in’ effects on alcohol and ‘crowding-out’ effects on food and health expenditure worsens household welfare in Ghana.

## INTRODUCTION

Globally, the prevalence rate of tobacco consumption was estimated at 23.7% in 2010^1^. The prevalence rate for Africa was estimated at 15.8% in 2010, the lowest compared to other continents^1^. Ghana’s estimated tobacco smoking prevalence rate of 5.3% in 2010 is not alarming compared to the global or the African value. However, the projection is that prevalence will increase in Ghana over the coming decades.

Annually, tobacco is estimated to result in the death of 5000 Ghanaians^2^. It is estimated that around 3900 males and 1092 females died from smoking-related diseases in 2016, which translates to 3.95% of male and 1.23% of female deaths attributable to tobacco use in 2016^2^.

Apart from the negative effects of tobacco use, in terms of morbidity and mortality, tobacco has crowding-in and crowding-out effects on household expenditure. The earliest study to investigate this was conducted by Efroymson et al.^3^ who estimated household expenditure patterns in Bangladesh. Some of the much earlier studies such as that of Barraclough^4^, pointed out the potential crowding-out of other household expenditure by tobacco and possible welfare implications on the household, but did not discuss these in detail. The earliest studies were based on simple comparison of means^3,4^. The findings revealed that food, health, housing, and education expenditure were displaced by tobacco^4,5^.

The challenge with making inferences about expenditure patterns from a simple comparison of means is that the analysis does not take into consideration differences in the demographic and socio-economic factors between smoking and non-smoking households, resulting in biased estimates.

Subsequently, the methodology evolved to use econometric models, which took into consideration socio-economic and demographic factors when estimating differences in the expenditure patterns of households. These studies were mainly conducted in China^6,7^, Cambodia^8^, Indonesia^9^ and the US^10^. They pointed out that expenditure on food was consistently displaced by tobacco. Other expenditures such as those on education, health, housing, savings, insurance, and farm productivity, were also found to be displaced by tobacco; while tobacco and alcohol were complements^6,10^.

A new wave of studies controlled for possible endogeneity in the demand functions used in estimating the expenditure patterns of households. This was necessary because the two variables ‘tobacco expenditure’ and ‘net-of-tobacco household expenditure’ are endogenous in the specification of demand functions^11,12^. Failure to control for endogeneity could lead to incorrect inferences about the impact of tobacco on other household expenditure^13^.

In order to address possible endogeneity in the econometric functions, subsequent studies used instrumental variables^11-16^. John^11^, in a study of India, used household expenditure as an instrument for net-of-tobacco household expenditure, following Vermeulen^17^. He also used the adult sex ratio, adult males to adults in the household, as an instrument for tobacco expenditure. Following Banks et al.^18^, Pu et al.^12^ also used household income as an instrument for net-of-tobacco household expenditure. Koch and Tshiswaka-Kashalala^14^, using a modified version of the Quadratic Almost Ideal Demand System developed by Banks et al.^18^, instrumented per adult equivalent net expenditure with per adult equivalent income and tobacco expenditure with a composite smoking prevalence rate. Chelwa and van Walbeek^13^ instrumented net-of-tobacco household expenditure with the value of household assets. San and Chaloupka^15^ instrumented tobacco expenditure with the adult female ratio. Jumrani and Birthal^16^ used peer-effect measures as an instrument for tobacco expenditure. The peer-effect measure is the average spending on tobacco or alcohol of a given household’s peer group (its village) net of the household’s own spending on that good.

The challenge with using some instruments is that they may violate the exclusion restriction. For instance, the adult sex ratio may be more associated with certain types of expenditure such as tobacco, alcohol and entertainment^6,10^ but less associated with other types of expenditure, such as those related to clean cooking fuels and the welfare of children^4,8^, as highlighted by Chelwa and van Walbeek^13^. The findings of studies that used instrumental variables to correct issues of endogeneity were similar to earlier findings that used simple comparison of means and basic econometric models.

The challenge with studies using instrumental variables to address endogeneity is that it is difficult to know whether the exclusion restriction has been met in practice^13,15,16^.

Chelwa and Koch^19^ avoided using instrumental variables but instead used Genetic Matching to expenditure quartiles to ascertain the effect of tobacco on other household expenditure. They found that food was crowded out by tobacco in the poorest households.

This study makes a contribution to the literature by estimating Engel curves using survey data from the 2012/2013 Ghana Living Standards Survey to ascertain whether tobacco’s crowding-out or crowding-in effects are also present in Ghana. Unlike previous studies, we use the GMM 3SLS method that results in efficient estimators that correct for heteroskedastic errors inherent in cross-sectional datasets of this kind.

## METHODS

Two methods are used, first we conduct t-tests on the difference in mean expenditure shares of tobacco-consuming and non-tobacco-consuming households to ascertain whether there are prima *facie* differences in household expenditure patterns. The t-tests will make use of sample survey weights to adjust for design elements of the survey^20^.

The analysis will further estimate Engel curves using the Quadratic Almost Ideal Demand System (QUAIDS) developed by Banks et al.^18^. The QUAIDS, which is consistent with consumer theory, allows for goods to be modeled as luxuries or necessities at certain income levels. Thus, it provides a more accurate representation of consumer behavior across income groups. The Engel curves will be estimated using the Generalized Method of Moment Three-Stage-Least-Squares (GMM 3SLS) with instrumental variables. Stata 15 was used to estimate the model.

The functional form below is implemented to estimate Engel curves for the various categories of expenditure *w_i_ = α_1i_ + α_3i_ q + α_4i_ a + β_1i_ (ln M) + δ_1i_ (ln M)^2^ + Ui* where w_i_ represents the budget shares (in percentages) of the *i* commodity group after deducting tobacco expenditure; *q* is the total expenditure on tobacco by a household; *a* is a vector of household characteristics, which include the age of the household head, the adult ratio, the logarithm of household size, average years of schooling of the entire household, rural/urban location, and the employment status of the household head; M is tobacco expenditure minus expenses on tobacco. *U_i_* is a random error, and *α* and *β* are coefficients.

Ordinarily, demand systems of the type estimated in this work ought to be estimated with prices, or, in the absence of price data, as is the case here, geographical fixed effects. We do not do this in this study. However, this is not much of a concern because tobacco prices have previously been argued to have limited cross-sectional variation in Ghana^21^.

Previous studies have found that the variables *q* and *M* are endogenous^11,13,15^. The Durbin-Wu-Hausmann test of the explanatory variables *q* and *M* in this study revealed that they are indeed endogenous. Instrumental variables are used to correct for endogenous variables. This study followed the literature^12,18^ and used household income as an instrument for M. The instrument for q was the adult sex ratio following the literature^11,12,13^. Adults are defined in this study as persons above 17 years of age. The prevalence rate for tobacco use in 2010 was 10.5% among adult males and 0.5% among adult females^22^.

The prevalence rate of tobacco use is higher among adults than non-adults and higher among adult males than adult females in Ghana^21,22^. Since more males than females smoke, the adult sex ratio is expected to be highly correlated with tobacco expenditure but unrelated to the error term.

The Breusch-Pagan/Cook-Weisberg test for heteroskedasticity revealed the presence of heteroskedastic errors. According to Wooldridge^23^, the GMM 3SLS is more efficient in the presence of heteroskedastic errors than the traditional 3SLS because it produces more efficient parameter estimates. The first stage regressions and F statistics revealed that total household income and adult sex ratio are valid and strong instruments for *M* and *q*, respectively. This study did not allow for the exclusion restriction to be violated in either of the instruments, as done by Chelwa and Van Walbeek^13^. In any case, they showed that the results are not sensitive to allowing for the instrument to be correlated with the error term^13^.

### Data

This study uses data from Round 6 of the Ghana Living Standards Survey conducted by the Ghana Statistical Service [http://www.statsghana.gov.gh/nada/index.php]^24^. The Ghana Living Standards Surveys are nationally representative surveys that consist of data at the individual, household, and community levels. Of interest to this study is the household section, which comprises data on housing characteristics, agricultural inputs, crop production, and expenditure on food items, assets, savings, and loans.

The Ghana Statistical Service has a complete list of enumeration areas based on previous censuses. The enumeration areas serve as the primary sampling units while households within each enumeration area serve as the secondary sampling units. A two-stage stratified random sampling design was employed. Enumeration areas were first stratified according to the ten administrative regions of the country and then according to rural and urban areas of location. The distribution of the selected enumeration areas in the ten regions was based on probability proportionate allocation using the population size.

The Ghana Living Standards Survey Round 6 enumeration exercise spanned one year from October 2012 to October 2013. In all, 1200 enumerative areas were considered at the first stage of sampling; 15 households were subsequently selected from each enumerative area. Round 6 yielded a sample of 16772 households. Of these 15528 households (92.62%) did not consume any tobacco; 7 households were deleted from the dataset for having zero value for annual household expenditure. Each household was revisited every 6th day in a 35-day cycle.

During the survey, a diary of daily expenditure was used to supplement the interviews. During the first visit, a literate member of the household was trained to record all subsequent expenditure and submit the diary to the interviewer on his next visit. Where a household had no literate member, the enumerator made daily visits to record all expenditure in the diary.

The Ghana Living Standards Survey reports expenditures separately for alcohol and different types of tobacco. The survey asked respondents how much they spent on each item per relevant time period. The information so obtained was aggregated annually. These items were later grouped under 12 broad categories of expenditure. These categories are: 1) Food and non-alcoholic beverages; 2) Alcoholic beverages; 3) Clothing and footwear; 4) Housing, water, electricity and gas; 5) Furnishings, household equipment and maintenance; 6) Health; 7) Transport; 8) Communications; 9) Recreation and culture; 10) Education; 11) Miscellaneous goods and services; and 12) Tobacco.

## RESULTS

### Descriptive statistics

Table 1 shows the descriptive statistics pertaining to 2012/2013. The data have been segregated into smoking and non-smoking categories. Table 1 shows that the average annual expenditure on tobacco by tobacco-consuming households was 123.86 GHS (100 Ghanaian Cedis about 53 US$ in 2012). The prevalence rate of tobacco use among households was 7.38%.

**Table 1:** Descriptive statistics from the 2012/2013 Ghana Living Standards Survey (N=16765)

*Description*	*Smoking households*	*Non-smoking households*	*Full sample*
**Average annual household expenditure (GHS)**	5104	7071	6926
**Median annual household expenditure (GHS)**	3805	5215	5109
**Average annual tobacco expenditure (GHS)**	123.86	0.00	9.14
**Average number of children in household**	2.96	1.95	2.02
**Average number of adult males**	1.42	1.03	1.06
**Average household size**	5.75	4.20	4.32
**Average number of adults in household**	2.79	2.25	2.29
**Average age of household head (years)**	49.47	45.55	45.84
**Average age of adults in household (years)**	41.64	39.41	39.58
**Average age of children in household (years)**	8.09	8.16	8.15
**Average years of education of household head (years)**	9.89	12.0	11.91
**Average years of education of most**			
**educated household member (years)**	9.41	12.05	11.88
**Percentage of households not consuming tobacco**			92.62
**Percentage of households consuming tobacco**			7.38

GHS: Ghanaian Cedis; 100 GHS about 53 US$ in 2012.

### Differences in mean expenditure shares

Table 2 shows the mean expenditure shares for tobacco-consuming and non-tobacco-consuming households for the full sample and by quintiles. Q1 is the poorest quintile while Q5 is the richest, by household income. The results of the t-tests on the difference in mean expenditure shares by tobacco-consuming and non- tobacco-consuming households are presented in Table 3.

**Table 2:** Average annual household expenditure share (%) in 2012/2013

*Expenditure*	*Income quintile*					*Full sample*
	*Q1*	*Q2*	*Q3*	*Q4*	*Q5*	
**Food**						
Non-smoker	57.40	57.42	56.06	53.34	49.05	54.56
Smoker	52.68	52.04	51.38	49.75	48.58	51.55
**Alcohol**						
Non-smoker	2.35	2.37	1.40	1.28	1.07	1.68
Smoker	5.94	6.90	4.62	4.82	4.00	5.62
**Clothing**						
Non-smoker	8.62	7.33	7.53	7.29	7.47	7.65
Smoker	7.99	6.34	8.33	7.43	7.24	7.52
**Housing**						
Non-smoker	12.21	12.35	11.94	12.46	14.02	12.61
Smoker	12.34	12.10	12.08	12.46	13.66	12.37
**Furnishings**						
Non-smoker	1.77	1.98	2.13	2.29	2.41	2.12
Smoker	1.70	1.68	1.96	1.98	2.07	1.81
**Health**						
Non-smoker	1.20	1.66	1.33	1.28	1.06	1.30
Smoker	1.64	2.05	1.70	1.77	1.65	1.77
**Transport**						
Non-smoker	4.28	4.36	4.97	5.85	7.23	5.37
Smoker	3.70	3.58	4.58	4.71	5.90	4.15
**Communications**						
Non-smoker	3.91	3.63	4.64	5.26	5.69	4.65
Smoker	2.45	2.49	3.08	3.80	3.87	2.87
**Recreation**						
Non-smoker	2.17	2.36	2.47	2.55	2.67	2.45
Smoker	3.03	3.14	2.62	2.71	2.65	2.91
**Education**						
Non-smoker	1.08	0.87	1.26	1.38	1.69	1.27
Smoker	0.66	1.04	1.49	1.54	1.47	1.08
**Miscellaneous**						
Non-smoker	4.99	5.67	6.27	7.01	7.64	6.34
Smoker	3.84	4.35	5.27	5.79	5.80	4.64
**Tobacco**						
Non-smoker	0.00	0.00	0.00	0.00	0.00	0.00
Smoker	4.03	4.28	2.89	3.24	3.11	3.71

**Table 3:** Difference in mean expenditure share (%) between non-smoking and smoking households in 2012/2013

*Expenditure*	*Income quintiles*					*Full sample*
	*Q1*	*Q2*	*Q3*	*Q4*	*Q5*	
**Food**	4.72^a^	5.38^a^	4.69^a^	3.59^a^	0.47	3.01^a^
**Alcohol**	-3.58^a^	-4.53^a^	-3.22^a^	-3.54^a^	-2.93^a^	-3.94^a^
**Clothing**	0.63	0.99	-0.80	-0.13	0.23	0.13
**Housing**	-0.13	0.25	-0.14	-0.01	0.36	0.24
**Furnishings**	0.07	0.30	0.17^b^	0.31	0.33	0.31^b^
**Health**	-0.44^a^	-0.39	-0.37^b^	-0.49^b^	-0.59^b^	-0.47^a^
**Transport**	0.58	0.78	0.39	1.14	1.33	1.22^a^
**Communications**	1.46^a^	1.13^a^	1.56^a^	1.46^b^	1.82	1.78^a^
**Recreation**	-0.86^a^	-0.79	-0.14	-0.16	0.02	-0.46^a^
**Education**	0.43^a^	-0.17	-0.23	-0.16	0.22^b^	0.19^a^
**Miscellaneous**	1.15^a^	1.32^a^	0.99	1.23	1.84	1.70^a^
**Tobacco**	-4.03^a^	-4.28^a^	-2.89^a^	-3.24^a^	-3.11^a^	-3.71^a^

A t-test that made use of the survey weights was used. The difference in weighted mean expenditure share produces differences in mean expenditure share between tobaccoconsuming and non-tobacco-consuming households using survey weights. A positive value indicates that the expenditure on this category by non-tobacco-consuming households is higher than the expenditure of tobacco-consuming households. a Implies the difference is statistically significant at the 1% level. b Implies the difference is statistically significant at the 5% level.

The results in Table 3 show differences in household expenditure in almost all the categories of household expenditure. However, large differences are observed in the categories of food, alcohol, and communications. Tobacco consumers tend to spend more on alcohol, while non-tobacco consumers tend to spend more on food and communications.

The difference in budget allocation to education between tobacco-consuming and non-tobacco-consuming households is rather small. This may be explained by the introduction of a 3 US$ per child capitation grant introduced by the government in 2005/2006 to help reduce the cost of education^25^.

### Regression results

Table 4 presents the results of the regression estimation of Engel curves.

**Table 4:** Results of the quadratic conditional Engel curve, 2012/2013 (N=13228)

*Factors*	*Food*	*Alcohol*	*Clothing*	*Housing*	*Furnishings*	*Health*	*Transport*	*Communications*	*Recreation*	*Education*
**q**	-39.8053^a^	21.9924^a^	2.4257	-10.5211^a^	0.2912	-3.0311^a^	9.0855^a^	16.8698^a^	9.4274^a^	1.8017
**ln M**	-28.9409	17.9318	8.8938	-70.7935^a^	0.5145	-10.9376^b^	9.2794	55.3674^a^	27.5191^a^	7.7681
**(ln M)^2^**	1.2301	-1.0928	-0.5413	4.2008^a^	-0.0049	0.6073^b^	-0.3512	-3.1303^a^	-1.5539^a^	-0.4338

Parameters of q are divided by 100.

a Shows levels of statistical significance at 1%.

b Shows levels of statistical significance at 5%.

The factor q shows the total pre-allocated expenditure on tobacco and it indicates the extent of crowding-out. For example, for every 1 GHS increase in the pre-allocated amount on tobacco, there is an increase by 0.22% in the budget share allocated to alcohol, i.e. 0.0022×*M* , where *M* is the annual budget (in GHS) of a given household after deducting tobacco purchases.

The factors (*ln M*) and (*ln M*)^2^ indicate whether households allocate more or less expenditure to an expenditure category as they become wealthier, which helps identify which expenditure categories households consider necessities, luxury, inferior, or sticky goods at different income levels.

The results show that tobacco expenditure crowds-in expenditure on alcohol, transport, communications and recreation, and crowds-out food, housing, and health at the 5% level of significance. A 10% increase (12.39 GHS) in tobacco expenditure leads to an increase in the budget for alcohol by 2.72%, transport by 1.13%, communications by 2.09%, and recreation by 1.17%. A 10% increase (12.39 GHS) in tobacco expenditure leads to a decrease in the budget for food by 4.93%, housing by 1.30%, and health by 0.38%.

## DISCUSSION

This study aims to contribute to the literature on the effect of tobacco on other household expenditure. The study used a GMM 3SLS to estimate Engel curves in order to ascertain the effect of tobacco on other household expenditure using the 2012/2013 Ghana Living Standards Survey. The results show a crowding-in of alcohol, transport, communications and recreation, and a crowding-out of food, housing, and health expenditure by tobacco. This is consistent with many findings in the literature, which found that tobacco consuming households spend less on areas that are more likely to improve the welfare and productivity of households^3,6,8^.

Lower expenditure on food by tobacco-consuming households compared to non-tobacco consuming households suggests a possible malnourishment of children as a large part of the food budget is diverted to tobacco consumption. Similar findings have been observed in Bangladesh^3^, Indonesia^9^, and Turkey^15^.

The findings further reveal a decrease in the quality of health of tobacco-consuming households as expenditure on health is crowded out and alcohol expenditure is crowded in. Other studies found that a similar effect on health expenditure contributed to the impoverishment of millions of tobacco consumers^7,26^.

### Limitations

A limitation of this study is that the data used is provided at the household level whereas expenditure decisions are often made at the individual level, with some aspects of intra-household bargaining taking place. Unfortunately, limitations within the dataset do not allow conducting the analysis at the individual level. Another limitation of the study is that often in the literature, specifications of the equation for w_i_, which represents the budget shares, allow for the inclusion of a dummy variable with interactions to test for preference heterogeneity following Vermeulen^17^. We do not do this in the present study because we would like to keep the demand system rather parsimonious. In any case, we believe that any heterogeneity in preferences between smoking and non-smoking households is adequately accounted for via the method of instrumental variables used in this study. Chelwa^27^ has made similar arguments in favor of a parsimonious demand system with instrumental variables estimation. Lastly, as noted in the methodology section, the analysis did not control for tobacco prices using, for example, geographical fixed effects. However, we believe that this is not a big concern because previous work has shown that there is limited cross-sectional variation in prices in Ghana^21^.

## CONCLUSIONS

This study highlights the fact that tobacco consumption is likely to lead to the deterioration of households’ welfare in Ghana. This is explained by the fact that tobacco consuming households spend more on alcohol, but less on food and health needs. Our results show that intra-household allocation in Ghana can benefit from the introduction of tobacco control policies.
